# Neurodevelopmental outcomes in individuals with VACTERL association. A population-based cohort study

**DOI:** 10.1371/journal.pone.0288061

**Published:** 2023-06-29

**Authors:** Ann-Marie Kassa, Helene Engstrand Lilja

**Affiliations:** 1 Department of Women’s and Children’s Health, Uppsala University, Uppsala, Sweden; 2 Department of Pediatric Surgery, University Children’s Hospital, Uppsala, Sweden; 3 Department of Women’s and Children’s Health, Karolinska Institutet, Stockholm, Sweden; 4 Department of Pediatric Surgery, Karolinska University Hospital, Stockholm, Sweden; Macau University of Science and Technology, HONG KONG

## Abstract

**Background:**

Studies on neurodevelopmental outcomes in individuals with congenital anomalies who undergo neonatal surgery are scarce and have reported contradictory findings based on small study groups. The congenital condition VACTERL association includes at least three malformations: vertebral anomalies, anorectal malformations, cardiac defects, tracheoesophageal fistula with or without esophageal atresia, renal anomalies and limb deformities. Most of these patients undergo surgery during their first days of life. Neurodevelopmental disorders include a broad group of disabilities involving some form of disruption to brain development. Attention deficit hyperactivity disorder (ADHD), autism spectrum disorders (ASD) and intellectual disability (ID) are diagnoses included in this group. The aim of the study was to investigate the risk of ADHD, ASD and ID in a cohort of individuals with VACTERL association.

**Method:**

Data was obtained from four Swedish national health registers and analyzed using the Cox proportional hazards model. Patients born 1973–2018 in Sweden with the diagnosis of VACTERL association were included in the study. For each case five healthy controls matched for sex, gestational age at birth, birth year and birth county were obtained.

**Results:**

The study included 136 individuals with VACTERL association and 680 controls. Individuals with VACTERL had significantly higher risk of ADHD, ASD and ID than the controls; 2.25 (95% CI, 1.03–4.91), 5.15 (95% CI, 1.93–13.72) and 8.13 (95% CI, 2.66–24.87) times respectively.

**Conclusions:**

A higher risk of ADHD, ASD and ID was found among individuals with VACTERL association compared to controls. These results are of importance to caregivers and to professionals participating in follow ups of these patients in providing early diagnosis and support, aiming to optimize the quality of life of these patients.

## Introduction

The congenital condition VACTERL association includes at least three malformations: vertebral anomalies (V), anorectal malformations (A), cardiac defects (C), tracheoesophageal fistula (TE) with or without esophageal atresia, renal anomalies (R) and limb deformities (L) [1]. Birth prevalence in Europe is recently estimated to be 6.25/100,000 [2] and accordingly less than 12 children with the diagnosis are likely to be born in Sweden per year. Most children with VACTERL association undergo neonatal surgery and often repeated procedures under anesthesia during childhood [1]. Usually, no cognitive impairments are linked to the diagnosis [3, 4].

Toxic effects on the brain after exposure to anesthetics in new-born animals have been reported [5–7]. After anesthesia with drugs often used in pediatric anesthetic procedures widespread neurodegeneration was found in the brain of newborn rats [7] and an increased cell death and inflammatory responses in the brains of neonatal piglets [6]. After combined surgery and anesthesia an increased cell death was reported in eight areas of the brain in piglets [8] and in the central nervous system in seven days old rats [9] compared to after only anesthesia. Negative impact from anesthesia has been observed on motor and socio-emotional aspects of behavioral development [10], memory and learning in various animal models [7, 11] with increased long-term cognitive impairment when surgery was added [9]. The development stage of the brain at exposure and the degree of exposure have been identified as important factors affecting the neurotoxicity of anesthetics [12]. Although these findings are difficult to transfer, [13, 14] concerns have been raised that human newborns could be at risk of neurodevelopmental dysfunctions after general anesthesia [15].

From systematic reviews an increased risk of impaired cognitive functions was reported in individuals with esophageal atresia (EA) [16], gastro-intestinal anomalies [17], non-cardiac congenital anomalies requiring surgery [18] and individuals undergoing cardiac surgery before six months of age [19]. A matched cohort study reported learning difficulties after repeated anesthesia and surgery before the age of two [20]. On the contrary no difference in IQ compared to siblings was found after exposure to surgery and anesthesia [21] and when comparing non- single- and multi-exposure to anesthesia before the age of three [22].

Population based studies have reported various neurodevelopmental outcomes such as increased risk of Attention Deficit Hyperactivity Disorder (ADHD) and Autism Spectrum Disorders (ASD) in individuals with anorectal malformations (ARM) [23], ADHD after repeated general anesthesia before age of three [24] and developmental delay and ADHD after minor surgery in anesthesia before the age of five [25]. In a large observational study children exposed to repeated procedures in anesthesia before the age of two had an increased risk of developing ADHD [26].

On the contrary no increased risk of neurodevelopmental impairment was found in a Randomized Controlled Trial where children were allocated to general or regional anesthesia for inguinal repair before the age of 60 weeks postmenstrual age [27] nor in population based studies after abdominal surgery during infancy [28] and in individuals born with Hirschsprung disease [29].

Neurodevelopmental disorders include a wide group of disabilities associated with disturbance in brain development. ADHD, ASD and Intellectual disability (ID) share common characteristics such as onset in childhood, being more common in males than in females, with high heritability and a high overlap between the disorders [30]. Genetic, prenatal and perinatal factors are included in the multifactorial etiology [30–33]. ADHD is characterized by inattention, motor hyperactivity and impulsivity [31] with a worldwide prevalence of approximately 5% in children and adolescents [34] and 2.5% in adult populations [35]. ASD with an approximate prevalence of 1.5–1.7% in developed countries implies impaired social communication and interaction and repetitive behavior [32, 36]. The definition of ID is reduced adaptive behavior and reduced intellectual function appearing in childhood or adolescence, confirmed through measured IQ ≤ ‒2 standard deviations (70 ±5) or through clinical assessment. Mild ID is defined as IQ range 50–69. More than half of the cases have genetic origin [33]. Prevalence is estimated to be 11/1000 [37].

Studies of neurodevelopmental outcomes after neonatal surgery in children with congenital anomalies are scarce and include small study populations with contradictory findings [16–19, 23, 29, 38–43]. Previous studies on the neurodevelopmental outcome in individuals with VACTERL are limited to a few publications with small sample sizes reporting on ID, developmental delay, intelligence, and attention [4, 44]. Our finding of attention difficulties in eight out of ten evaluated children aged 5–7 years with VACTERL association [44] motivated a larger population based register study to investigate the prevalence of ADHD and also the diagnoses of ASD and ID as being included in the group of neurodevelopmental disorders associated with disturbance in the brain development.

The aim of this study was to investigate the prevalence and risk of being diagnosed with ADHD, ASD and ID in a national population-based cohort of individuals with VACTERL association.

## Methods

The study was approved by the Swedish Ethical Review Authority, registration number 2019–06506, and amendments 2021–00777 and 2021–04067. Since all data were anonymized, the Swedish Ethical Review Authority approved that no informed consent was obtained from the study group.

The Swedish National Board of Health and Welfare administers national registers enabling analysis and follow up of the Swedish healthcare services [45]. Since the registers are based on personal identification numbers it is possible to combine information from various registers. Data was collected from the following registers.

**Fig 1:**
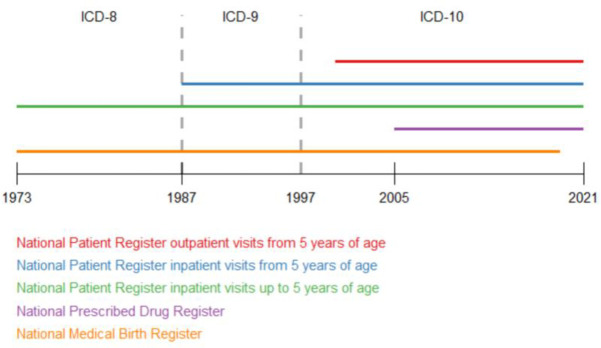
Timeline for data collection from Swedish national health registers.

The National Patient Register (NPR) contains information starting from 1964 and since 1987 includes information of all in-patient care in Sweden. From 2001, information about outpatient physician visits including day surgery and psychiatric care is also registered [45]. High validity of 85–95% Positive Predictive Value of inpatient diagnoses has been reported [46]. The Medical Birth Register (MBR) was founded in 1973 and includes data on all deliveries in Sweden. Missing data on deliveries is estimated to be 1–3% during the latest 20 years. The Cause of Death Register (CDR) contains information on death causes since 1961 and since 1991 includes data on all deceased persons. Missing data on death causes is estimated to be 1–2% during recent years. The Prescribed Drug Register (PDR) started in July 2005 and contains information on all prescribed drugs dispensed through a pharmacy and is estimated to be of a high quality since the registration process is automated [45]. Data was collected from these registers for the time periods displayed in Fig 1.

### Study cohort

The study group included patients born in 1973–2018 identified through the diagnosis codes from the Swedish versions of International Statistical Classification of Diseases and Related Health Problems (ICD) in ICD-8 for 1973–1986, ICD-9 for 1987–1996, and ICD-10 from 1997. Inclusion and exclusion criteria are presented in Table 1.

**Table 1:** Inclusion and exclusion criteria of the study group.

Inclusion criteria	Exclusion criteria
Patients born in 1973–2018 identified through ICD-codes in ICD-8 for 1973–1986 ICD-9 for 1987–1996 ICD-10 from 1997	
At least one of the following inclusion criteria registered up to and including the five-year birthday • ICD-code for VACTERL association: • ICD-10: Q872, ICD-9: 759H 759W • A combination of ≥ 3 ICD-codes for congenital malformations: Vertebral malformations: ICD-10: Q76.4W ICD-9: 756B ICD-8: 756,18 756,19 Anorectal malformations (ARM): ICD-10: Q42.0-Q42.3 Q43.7 ICD-9: 751C 751F ICD-8: 751,20–751,25 751,29 751,52 Cardiac malformations: ICD-10: Q20-Q24 ICD-9: 745A -745F 745W 745X, 746A-746X ICD-8: 746,09–746,84 747,19–747,39 Esophageal atresia (EA): ICD-10: Q 39.0 Q39.1 Q39.2 ICD-9: 750D ICD-8: 750,20 750,28 Renal malformations: ICD-10: Q60-Q64 ICD-9: 753A 753C 753D ICD-8: 753,00–753,02 753,20–753,39 Limb malformations: ICD-10: Q71 Q72 ICD-9: 755C 755 D ICD-8: 755,20–755,39	
	ICD-codes indicating Chromosomal aberrations ICD-10: Q90-Q99 ICD-9: 758A-758X ICD-8: 759,30–759,59 Other syndromes ICD-10: Q870 Q871 Q873-Q89 (including CHARGE syndrome Q878)
Additional inclusion criteria • Verification of an ICD-code of ARM with a surgical procedure code specific for ARM **or** one hospital admission of ≥ 4 four consecutive days with ARM diagnosis during the first three months of life • Verification of an ICD-code of EA with a surgical procedure code specific for EA **or** one hospital admission of ≥ 7 consecutive days with EA diagnosis during the first 30 days of life≥• 1 day hospital stay during first year of life	

### Control cohort

A control group of five healthy individuals per included case, matched for sex, gestational age at birth, birth year and birth county was collected from the MBR. Individuals with the exposure of the above mentioned inclusion diagnoses, were excluded through linkage with the NPR. As for the cohort, exclusion for chromosomal aberrations and syndromes were made.

### Outcome variables

Data of all in-patient hospital visits up to and including the five-year birthday was collected from the NPR. From the age of five, outcome diagnoses from ICD-9 and ICD-10 including the age at first registration were obtained for ADHD ICD-10: F90.0-F90.1, F90.8-F90.9, ICD-9: 314, ASD ICD-10: F84, ICD-9: 299 and ID ICD-10: F70-F79, ICD-9: 317–319. Information on the Anatomical Therapeutic Chemical (ATC) codes of prescribed drugs for treatment of ADHD specified as N06B and C02AC02 was obtained from the PDR and information of deceased individuals from the CDR.

### Data analysis

Categorical variables are presented as numbers (n) and percentages (%) and continuous variables as median (M) and range. For significance testing of differences between cases and controls in numerical variables, Mann-Whitney U-test was applied. Chi-square test was used to test differences in proportions when expected values in each cell were higher than five, Fisher’s exact test was otherwise applied. The differences in outcome diagnoses were analyzed with time-to-event analysis using the Cox proportional hazards model where the hazard ratio (HR) indicates the relative difference in the risk of the outcome between case and control groups. The significance level was set to p<0.05. For the statistical analyses R version 4.1.1 (R Foundation for Statistical Computing, Vienna, Austria) was used.

## Results

**Fig 2:**
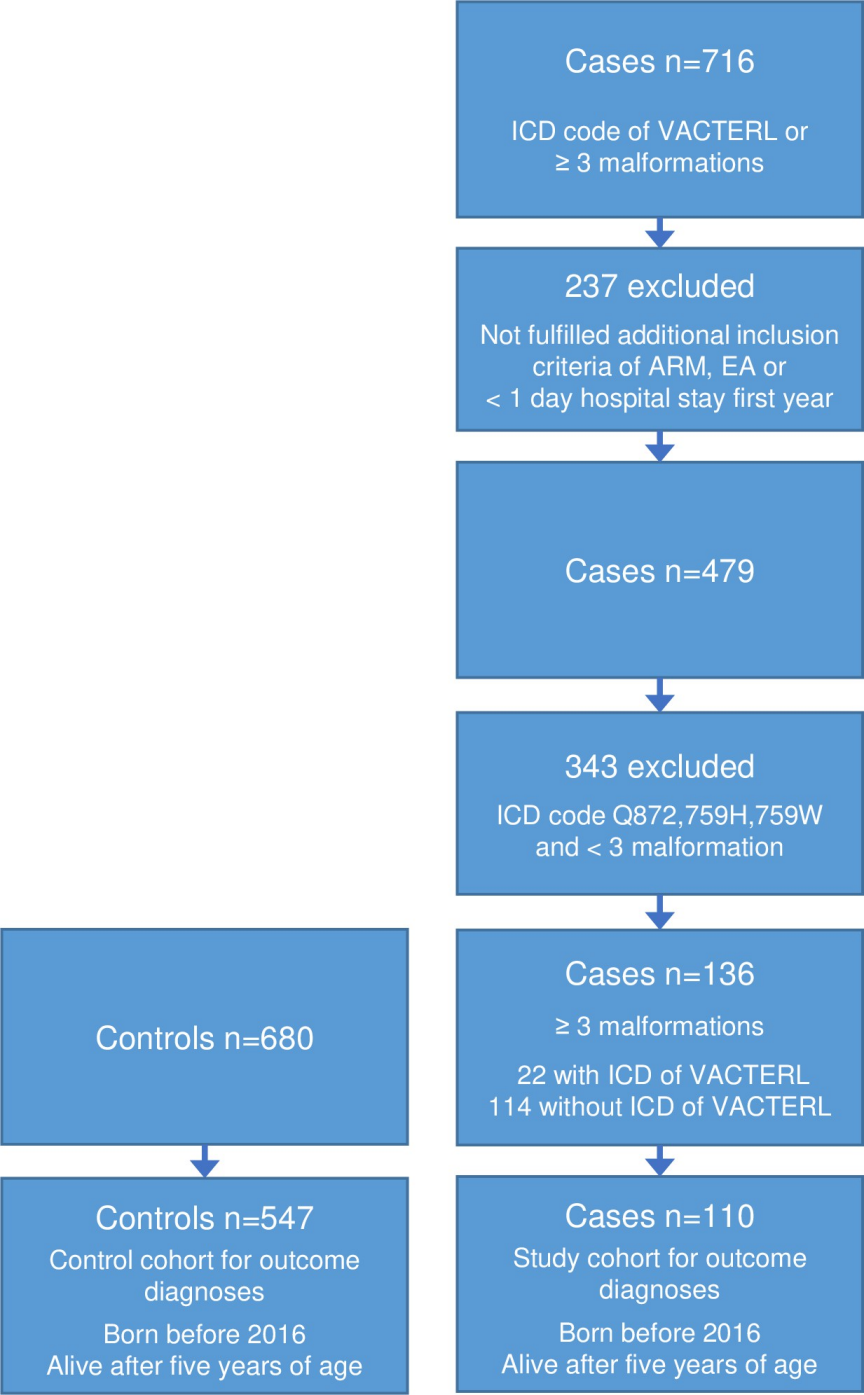
Flowchart of included cases and controls. Individuals included after applied exclusion criteria of ICD-codes of chromosomal aberrations and other syndromes.

After applying the exclusion criteria, 716 individuals with an ICD-code of VACTERL association or ICD-codes of at least three of the malformations included in VACTERL association were identified. The application of the additional inclusion criteria resulted in 479 individuals. Among them 343 only had the diagnosis code of Q872, 759H or 759W and none or less than three of the listed malformations and were therefore not included. The final study group consisted of 136 individuals with at least three of the listed malformations whereof 22 with and 114 without the ICD code for VACTERL association. The matched control group consisted of 680 individuals. Flow chart of included cases and controls is displayed in Fig 2.

Characteristics of the study groups are presented in Table 2.

**Table 2:** Characteristics of VACTERL association cohort and controls.

Variable	VACTERL N = 136	Control N = 680	
Sex			
Male, n (%)	73 (53.7%)	365 (53.7%)	
Female, n (%)	63 (46.3%)	315 (46.3%)	
Gestational age, Md (range)	38 (28–43)	38 (28–43)	
Prematurity <37 weeks, n (%)	44 (32.4%)	220 (32.4%)	
Small for gestational age (SGA), n (%)	32 (25.2%)	28 (4.6%)	p <0.0001^a^
Vertebral malformation, n (%)	45 (33.1%)	1 (0.1%)	
Anorectal malformation, n (%)	83 (61.0%)	0 (0.0%)	
Cardiac malformation, n (%)	105 (77.2%)	2 (0.3%)	
Esophageal atresia, n (%)	55 (40.4%)	0 (0.0%)	
Renal malformation, n (%)	90 (66.2%)	0 (0.0%)	
Limb malformation, n (%)	48 (35.3%)	0 (0.0%)	
Hospital stay until one year of age (days), Md (range)	54 (2–365)	0 (0–112)	
Hospital stay until five years of age (days), Md (range)	71 (2–645)	0 (0–126)	
Individuals undergoing surgery within five years, n (%)	115 (84.6%)	0 (0.0%)	
Mortality, n (%)	17 (12.5%)	5 (0.7%)	p <0.0001^b^
Number of years of follow up, Md (range)	23.5 (3–48)	23.5 (3–48)	

Md: Median

^a^ Chi-square test

^b^ Fisher’s Exact test

In the study group, the median days of hospital stay until one year of age was 54 days (2–365) and during the first five years, 71 (2–645). The mortality rate in the VACTERL group was 12.5% compared to 0.7% in the control group (p < 0.0001) and age at death 0 (0–37) and 0 (0–46) respectively. When dividing the study cohort into two time periods according to the birth years, the mortality rate during 1973–1996 for VACTERL patients was 20.0% and for controls 1.5% (p<0.0001). During 1997–2018 the mortality rate was 5.6% for VACTERL patients and 0% for controls (p = 0.0007). In Table 3 the outcome and age at first registered outcome diagnosis are presented. Individuals born 2016 or later or deceased within five years were excluded, leaving 110 cases and 547 controls for the analysis. In the study cohort ADHD was found in 9 (8%) individuals, ASD in 8 (7.3%) and ID in 8 (7.3%) individuals whereof half with mild ID. There were no significant differences found between the age at first diagnosis between the two cohorts (Table 3).

**Table 3:** Outcome of diagnoses and median age at first diagnosis.

Variable	VACTERL n = 110		Control n = 547		
	n (%)	Age at diagnosis Md ^5^ (range)	n (%)	Age at diagnosis Md (range)
ADHD ^1^	9 (8.2%)	12 (8–22)	21 (3.8%)	18 (5–33)
ASD ^2^	8 (7.3%)	14.5 (8–32)	8 (1.5%)	14.5 (5–38)
ID ^3^	8 (7.3%)	16 (6–36)	5 (0.9%)	12 (6–24)
Mild ID ^4^	4 (3.6%)	17 (15–20)	4 (0.7%)	14.5 (10–24)

^1^ ADHD Attention deficit hyperactivity disorder

^2^ ASD Autism spectrum disorders

^3^ ID Intellectual disability

^4^ Mild ID Mild intellectual disability, also included in ID

^5^ Md: Median

Among the nine individuals in the study group diagnosed with ADHD, six (66.7%) had been prescribed ADHD medication, ATC codes C02AC02 or N06B, since the start of PDR.

After adjustment for sex, birth year and gestational age, the result of Cox regression indicated a significant Hazard Ratio of 2.25 (95% CI 1.03–4.91) times increased probability to be diagnosed with ADHD, 5.15 (95% CI 1.93–13.72) times with ASD, 8.13 (95% CI 2.66–24.87) times with ID and 5.11 (95% CI 1.28–20.44) times with mild ID compared to controls.

When dividing the cohort into two time periods, before and from 1997 when ICD-10 was implemented, an increased HR was observed in the cohort born between 1973 and 1996 for ADHD, ASD and ID compared to controls but not in the cohort born 1997 and later. The HRs for the outcome of diagnoses in the whole cohort are presented in Table 4 and as divided into two time periods in Table 5.

**Table 4:** Cox regression for outcome diagnoses in individuals born 1973–2015.

Outcome			Unadjusted HR ^5^ (95% CI)	P-value (unadjusted)	Adjusted HR ^6^ (95% CI)		P-value (adjusted)
	VACTERL n = 110	Controls n = 547					
ADHD^1^	9 (8.2%)	21 (3.8%)	2.27 (1.04–4.97)	0.0392	2.25 (1.03–4.91)	**0.0423**	
ASD ^2^	8 (7.3%)	8 (1.5%)	5.22 (1.96–13.92)	0.0010	5.15 (1.93–13.72)	**0.0011**	
ID ^3^	8 (7.3%)	5 (0.9%)	8.22 (2.69–25.13)	0.0002	8.13 (2.66–24.87)	**0.0002**	
Mild ID ^4^	4 (3.6%)	4 (0.7%)	5.18 (1.29–20.71)	0.0201	5.11 (1.28–20.44)	**0.0212**	

^1^ ADHD: Attention deficit hyperactivity disorder

^2^ ASD: Autism spectrum diagnoses

^3^ ID: Intellectual disability

^4^ Mild ID: Mild intellectual disability, also included in ID

^5^ HR: Hazard Ratio

^6^ Adjusted HR adjusted for sex, gestational age and birth year

**Table 5:** Cox regression for outcome diagnoses divided in individuals born 1973–1996 and 1997–2015 respectively.

Outcome			Unadjusted HR ^5^ (95% CI)	Adjusted HR ^6^ (95% CI)
**Individuals born 1973–1996**				
	VACTERL n = 56	Controls n = 277		
ADHD^1^	6 (10.7%)	12 (4.3%)	2.72 (1.02–7.24)	2.69 (1.01–7.17)
ASD ^2^	6 (10.7%)	4 (1.4%)	8.05 (2.27–28.56)	8.12 (2.29–28.81)
ID ^3^	6 (10.7%)	3 (1.1%)	10.37 (2.59–41.45)	10.33 (2.58–41.30)
Mild ID ^4^	4 (7.1%)	3 (1.1%)	6.94 (1.55–31.02)	6.89 (1.54–30.84)
**Individuals born 1997–2015**				
	VACTERL n = 54	Controls n = 270		
ADHD	3 (5.6%)	9 (3.3%)	1.71 (0.46–6.31)	1.68 (0.45–6.21)
ASD	2 (3.7%)	4 (1.5%)	2.51 (0.46–13.72)	2.47 (0.45–13.53)
ID	2 (3.7%)	2 (0.7%)	4.90 (0.69–34.76)	4.83 (0.68–34.31)
Mild ID	0 (0.0%)	1 (0.4%)		

^1^ ADHD: Attention deficit hyperactivity disorder

^2^ ASD: Autism spectrum diagnoses

^3^ ID: Intellectual disability

^4^ Mild ID: Mild intellectual disability, also included in ID

^5^ HR: Hazard Ratio

^6^ Adjusted HR adjusted for sex, gestational age and birth year

When adjusting also for SGA in the Cox regression model the results indicated significant Hazard Ratios for ADHD 2.56 (95% CI 1.12–5.84), ASD 3.52 (95% CI 1.16–10.66), ID 12.09 (95% CI 3.58–40.89) and Mild ID 7.27 (95% CI 1.55–34.05).

## Discussion

To our knowledge this is the first population-based study investigating the risk of being diagnosed with neurodevelopmental impairments in a cohort of individuals with VACTERL association. We found a significantly increased probability of being diagnosed with ADHD, ASD and ID in patients with VACTERL association.

In the literature there are conflicting data as to whether and how early surgery and anesthesia affect the brain during infancy [27, 28] and the first few years of life [20–22, 24–26]. Studies including individuals with various congenital anomalies undergoing neonatal surgery report contradictory findings of neurodevelopmental outcomes [16–19, 23, 29, 38–43]. Important factors for the neurotoxicity of anesthesia in newborn animals have been identified as the developmental stage of the brain and the degree of exposure [12]. Furthermore, combined surgery appears to cause additional cell death [8, 9]. Individuals with VACTERL association undergo at least one surgery during their first days in life, and often also repeated procedures in anesthesia with or without surgery during their first years in life. Since neurodevelopmental disorders are associated with some disturbance in brain development [30] our hypothesis was that these early exposures could increase the risk of neurodevelopmental disorders in patients with VACTERL association. Increased understanding of neurodevelopmental disorders such as ASD, ADHD and ID in patients with VACTERL association is crucial to support these children and their parents to thus enable good health-related quality of life.

Reports in previous literature on associations between the diagnosis of VACTERL and neurodevelopmental disorders are rare. Among ten 5-7-year-old children with VACTERL association, intelligence was within normal range while attention difficulties were found in eight children whereof two were subsequently diagnosed with ADHD [44]. In a study of 17 individuals with VACTERL association, an increased frequency of ID and mild developmental delay was found in a subgroup of individuals with congenital anomalies outside the scope of VACTERL association such as CHARGE syndrome [4]. In the present study, individuals with chromosomal aberrations and other syndromes were excluded.

The risk of ADHD in individuals with VACTERL association in our study was increased. Similarly, an increased risk of ADHD was reported in individuals with ARM [23] and with complex congenital heart disease who underwent surgery in early infancy [38]. Furthermore, an increased risk of ADHD was reported after single surgery before the age of five [25] and repeated anesthesia before two [26] and three years of age [24]. On the contrary, no increased risk of ADHD was found among individuals with Hirschsprung disease [29] or after abdominal surgery during infancy [28].

The risk of ASD in the VACTERL cohort was increased and even higher than the risk of ADHD. An increased risk of ASD has also been found among individuals with ARM [23], congenital diaphragmatic hernia (CDH) [39] and giant omphalocele [40] undergoing neonatal surgery like that for individuals with VACTERL association. Moreover, a population-based study reported associations between ASD and birth defects such as gastro-intestinal, genitourinary, and musculoskeletal and multiple anomalies [47]. Low birth weight and born small for gestational age (SGA) have in other studies been associated with ASD [48, 49]. A significantly higher proportion in the VACTERL group in our study were born SGA compared to controls. However, since the etiology is multifactorial it is difficult to evaluate the impact of SGA on the outcomes, also after controlling for sex and prematurity. In our study we found an increased risk of the chosen outcome diagnoses independent of including SGA or not in the Cox proportional hazards model. Thus, we conclude that occurrence of SGA does not explain the increased risk of these diagnoses in our study cohort. As Lampi et al. stated, SGA might be an indicator of other risk factors associated with ASD such as various complications during pregnancy [48].

In line with some other reports in children with major congenital anomalies, we found an increased risk of ID in the VACTERL cohort. ID has been reported in 4.8% of children with major non-cardiac congenital anomalies [42], 25% with EA, 20% with ARM [41] and 14–18% of children with CDH [39, 41]. In contrast, normal intelligence has been reported in cohorts of children with gastroschisis and EA [43, 50].

According to the literature, there is a high overlap between the three diagnoses of ADHD, ASD and ID [30]. High comorbidity in individuals with ADHD with other neurodevelopmental disorders has been reported such as ASD and ID [31] with reports of 20–50% for ASD [51]. Comorbidity in ASD has been reported in 40–70% for ADHD [52] and 11–65% for ID [32, 33, 36]. Some of the individuals in our study might contribute to more than one diagnosis but still the study indicates the risk of an outcome of neurodevelopmental disorder compared to controls.

In the present study we found no increased risk of ADHD, ASD or ID in individuals with VACTERL association born 1997–2015. We speculate that the neurodevelopmental outcomes together with lower mortality in the later cohort may reflect some improvements achieved in anesthetic and surgical methods and neonatal intensive care during the latest decades [1, 14]. However, it should be taken into consideration that the study group born 1997–2015 had a shorter follow-up period than the cohort with individuals born 1973–1996. To some extent this potential bias could be compensated for through the matched controls with the same follow-up periods. Additionally, in Sweden, neurodevelopmental diagnoses have lately been much discussed in the society and media, which has probably increased awareness of the signs of the disorders. Consequently, these disorders may have been diagnosed at an earlier age during recent decades.

Additionally, the general health outcomes in the VACTERL cohort may have improved during the latest decades due to progress in surgical and medical care. This is reflected in the decreasing mortality rate which was 20% among those born until 1996 compared to 5.6% among those born 1997 and later.

### Methodological considerations

A strength of the study was that it investigated neurodevelopmental outcome in patients with VACTERL association including the until now largest study population with the longest follow up. Another strength is the design of a population-based cohort study with data prospectively collected and with a control cohort matched on sex, gestational age at birth, birth year and birth county, thus reducing the risk of selection bias. Moreover, we included only individuals with at least three of the malformations VACTERL association encompasses, to avoid misclassification.

A limitation of the study was the inherent design of a register study since the data was not confirmed through review of medical records. Furthermore, it was not possible to control for various prenatal, perinatal and neonatal cofounding factors such as heredity, complications and risk behavior during pregnancy and low birth weight. Our aim was to match the controls also for SGA but unfortunately it resulted in considerably fewer controls.

To avoid misclassification we considered the ICD codes for VACTERL association not to be enough since there are other syndromes classified with the same ICD code. Thus, to make sure to obtain data on factual VACTERL patients we decided to include the ICD codes of at least three of the included malformations.

## Conclusions

A higher risk of ADHD, ASD and ID was found among individuals with VACTERL association compared to controls. These results are of importance to caregivers and professionals participating in the follow up of patients with VACTERL association in providing early diagnosis and support and thus optimizing the quality of life of these patients. Future prospective studies with long term follow up of individuals with VACTERL association are needed to increase knowledge of neurodevelopmental disorders in these patients.
